# Transcriptomic Dysregulation in Animal Models of Attention‐Deficit Hyperactivity Disorder and Nicotine Dependence Suggests Shared Neural Mechanisms

**DOI:** 10.1002/brb3.70444

**Published:** 2025-03-26

**Authors:** Sarah Van Horn, Heather Driscoll, Donna J Toufexis

**Affiliations:** ^1^ Patrick Leahy Honors College University of Vermont Burlington Vermont USA; ^2^ Vermont Biomedical Research Network Burlington Vermont USA; ^3^ Department of Biology Norwich University Northfield Vermont USA; ^4^ Department of Psychology University of Vermont Burlington Vermont USA

**Keywords:** attention‐deficit hyperactivity disorder, differential gene expression, pathway enrichment analysis, drug addiction, MAP kinase, nicotine, synaptic transmission

## Abstract

**Introduction:**

Attention‐deficit‐hyperactivity disorder (ADHD) is highly heritable and increases the likelihood of nicotine dependence (ND). The self‐medication hypothesis of nicotine use in ADHD proposes that ADHD patients seek nicotine for its ability to improve their symptoms, and they have less success quitting, possibly due to the worsening of ADHD symptoms in withdrawal.

**Methods:**

The present analysis compared transcriptomic data from the brains of rodent models of ADHD and those of ND, with a focus on striatal gene expression. Differential expression analysis, pathway enrichment analysis, and gene‐network mapping identified signaling networks and candidate genes that may contribute to the high co‐occurrence between ADHD and ND.

**Results:**

We identified novel differentially expressed genes (*PRKAG2*, *MAPK1*), and genes with known associations to either ADHD or ND (*ANK3*, *CALD1*, *CHRNA4*, *CHRNA7*, *CMTM8*, *DLG4*, *DUSP6*, *GNG3*, *GNG11*, *GRIK5*, *GRINA2, GRM5*, *ICAM2*, *KCNJ6*, *PRKAB1*, *SNAP25*, *SYNPO*, *SYT1, VAMP2*). In addition, synaptic transmission (hsa04728, R‐HAS‐112315, R‐HSA‐442755) and MAPK signaling pathways (hsa04010, hsa04014, hsa04015, R‐HSA‐5673001, R‐HSA‐5684996) were enriched in both ADHD and ND.

**Conclusion:**

The signaling pathways implicated by this analysis mediate neurological mechanisms known to contribute to ND. The association of analogous differently expressed genes and common signaling pathways suggests an important causal relationship between ND and ADHD that may be clinically important.

## Graphical abstract

Around 50% of people with attention‐deficit hyperactivity disorder (ADHD) develop nicotine dependence (ND); however, the mechanism for this relationship is unknown, and current treatments for ADHD do not prevent or slow the onset of ND. Transcriptomic analysis of the striatum and whole brains of three rodent models of ADHD and two of ND supports a role for mitogen activating protein kinase and upstream neurotransmitter systems in both disorders.

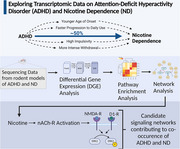

## 1 Introduction

### 1.1 Co‐Occurrence of Attention‐Deficit Hyperactivity Disorder (ADHD) and Nicotine Use Disorder (Nicotine Dependence [ND])

Although 8%–12% of the world has ADHD and twin studies suggest it is highly (76%) heritable, the disorder lacks an equivocal pattern of inheritance (Faraone et al. 2005). Childhood ADHD patients are more likely to smoke in adolescence and adulthood, are overall at least twice as likely to smoke, transition faster from infrequent to daily smoking, and have less success quitting (Wilens et al. 2002; Mitchell et al. 2018; Molina et al. 2018), suggesting ADHD and the proclivity to use certain drugs may be mechanistically related.

According to most interpretations, ADHD is a disorder of inhibitory control affecting motor activity, attention, working memory, and motivational regulation (Barkley 1997; Parry and Douglas, 1983; Sagvolden et al. 2005; Shelton and Barkley 1995). Addiction is a disease that overrides the brain's reward centers and prevents the inhibition of motivated behaviors (Moeller et al. 2016). Poor inhibitory control predicts SUD susceptibility in the general population (Jentsch and Pennington 2014). Impaired inhibitory control mechanisms likely contribute to the co‐occurrence of ADHD and SUD.

ADHD patients describe using nicotine to self‐medicate and report that it reduces ADHD symptoms and improves mood (Gehricke et al. 2009; Liebrenz et al. 2014). Some research suggests that nicotine uniquely modulates behavioral inhibition in ADHD patients, as it improves cognitive performance in adolescents/young adults with ADHD but not controls (Potter and Newhouse 2004, 2008, 2004, 2008; Potter et al. 2012). However, smokers with ADHD have less success quitting than controls (Mitchell et al. 2018) and more severe withdrawal symptoms (Bidwell et al. 2018; McClernon et al. 2011). Importantly, adverse health outcomes are more prevalent in ADHD patients due to poor lifestyle choices, including smoking (Wilens et al. 2002).

### 1.2 Genomic Evidence for ADHD

The heritability of ADHD is associated with familial psychiatric illness, specifically PTSD and substance use (Spencer et al. 2015). Genome‐wide association studies (GWAS) compare entire genomes and identify significant differences in DNA sequences between populations. GWAS has identified single nucleotide polymorphisms (SNPs) on or near genes related to dopaminergic and glutamatergic transmission, neuronal plasticity, and development in ADHD patients (Demontis et al. 2023). However, these findings do not provide insight into the effects of these SNPs nor do they indicate gene expression.

### 1.3 Brain Regions in ADHD and ND

The catecholamine hypothesis of ADHD suggests that dysregulation in catecholamine systems, particularly dopamine (DA) and norepinephrine (NE) systems, leads to the behavioral characteristics of ADHD. ADHD medications like amphetamines and methylphenidate target DA and NE reuptake transporters in support of this hypothesis. Compared to other regions implicated in ADHD, the striatum receives the most dopaminergic projections. MRI and fMRI studies repeatedly reveal reduced gray matter volume and function in the striatum of ADHD patients (Hoogman et al. 2017; Cupertino et al. 2020). These differences are present in the dorsal striatum, containing the caudate and putamen, and the ventral striatum, containing the nucleus accumbens (NAc). PET studies in ADHD patients that investigate DA reuptake transporter and receptor density show decreases in the striatum that negatively correlates with symptom severity (Volkow et al. 2009). Impaired frontostriatal connectivity is also associated with inhibitory control defecits in ADHD (e.g., Casey et al. 1997; Cubillo et al. 2010; Nikolaidis et al. 2022).

In ADHD, decreased DA activity in the striatum relates to symptom severity and impairment. These patients also exhibit disruptions in reinforcement processes influenced by striatal DA (see Kollins and Adcock 2014). Generally, ND develops from learning to seek nicotine rewards and is dependent on the frontostriatal reward circuit (Ortells and Arias 2010). The frontostriatal circuit connects the prefrontal cortex (PFC), responsible for cognitive control, to the striatum, which drives reward‐seeking. The short‐term rewarding effect of nicotine results from nicotine's agonism of nicotinic acetylcholine receptors (nAChRs) on DA neurons in the ventral tegmental area (VTA) that project to the NAc (L. Liu et al. 2012). Nicotine also affects DA neuron activity by modulating glutamatergic innervation of the VTA in a circuit with the PFC that contributes to long‐term nicotine reward (Mansvelder and McGehee 2000). Nicotine desensitizes nAChRs on gamma‐aminobutyric acid (GABA) neurons in a disinhibitory circuit that also increases DA release onto the striatum (Klink et al. 2001). Overall, nicotine increases DA activity in the striatum, which is the same region where deficient DA signaling is implicated in ADHD.

### 1.4 Using Rodent Models for Transcriptomic Analysis

RNA‐Seq is a technique that utilizes next‐generation sequencing to determine mRNA abundance in a tissue sample. The tissue must be preserved as a fresh‐frozen sample making this technique useful for lab animals but unsuitable for humans. Fortunately, several rodent models of ADHD have been developed based on phenotypic behavior, and RNA‐Seq analysis of these rodents may reveal transcriptional alterations that contribute to ADHD in humans.

In constructing rodent models for ADHD, researchers seek to replicate behavioral characteristics that define the disorder in human subjects and establish the efficacy of drug treatments for human ADHD in reducing these symptoms (Tripp and Wickens 2012). Selective breeding experiments have developed several rodent models of ADHD, and the spontaneously hypertensive rat (SHR) is the most widely used. Like children with ADHD, SHRs lose interest in delayed reinforcers much faster than their controls, the Wistar Kyoto rat (WKYs), and are more impulsive (Pardey et al. 2009; Sutherland et al. 2009; Bizot et al. 2007). These are the only rodent models of ADHD with publicly available RNA‐Seq data of the brain.

### 1.5 Rationale for the Present Experiment

Understanding gene expression patterns in rodent models of ADHD may reveal biological pathways related to behavior. Comparing the transcriptome of rodent models for ADHD to rodents with ND phenotypes may reveal differential expression patterns contributing to shared behavioral characteristics between the two disorders. Behaviorally, people with ADHD or ND show similar deficits in striatum‐dependent tasks. The striatum is the brain's center for reward processing and contributes to impulsivity and inhibitory control. Smokers show higher impulsivity, as they discount the value of delayed rewards at a steeper rate than non‐smokers, as people with ADHD, compared to controls (i.e., Baker et al. 2003; Amlung and MacKillop 2014; Jackson and MacKillop 2016; Marx et al. 2021). Inhibitory control deficits, assessed by the Go/No‐Go and continuous performance tasks, are also apparent in ND and ADHD (i.e., Bickel et al. 1999; Nikolaidis et al. 2022). The similarities between ADHD patients and smokers on striatum‐dependent behavioral tasks support the investigation of this region in the two disorders.

This investigation re‐analyzed publicly available RNA‐Seq and microarray data from the brains of rats and mice modeled after ADHD and ND to investigate commonly dysregulated features. The striatum was the central region examined, given its relevance to the development of ND and impairment in ADHD. The purpose of the analysis is to (a) survey global expression and genetic changes for ADHD and ND and (b) perform pathway analysis to identify biological mechanisms that may underscore the co‐occurrence of ADHD and ND phenotypes.

## 2 Methods

### 2.1 Dataset Search

NCBI Gene Expression Omnibus (Barrett et al. 2013; https://www.ncbi.nlm.nih.gov/geo/), or GEO contains public functional genomics data and was searched for RNA‐Seq datasets from brains of rodent models of ADHD and ND. For ADHD models, search terms included “Attention‐Deficit Hyperactivity Disorder,” “brain,” “mouse,” or “rat,” and two relevant RNA‐Seq datasets were identified; Sorokina et al. (2018, GSE116752) and Nakano et al. (2023, GSE211982). NCBI GEO also returned a microarray study that analyzed the whole brain of the SHR, the most used rodent model for ADHD (Yoshida et al. 2014, GSE41452). NCBI GEO was searched for rodent experiments with RNA‐Seq data from nicotine‐dependent rodents with search terms including “nicotine,” “brain,” “mouse,” or “rat.” Two relevant publications were identified; Yang et al. (2017, GSE89899) and Kozlova et al. (2021, GSE157683).

### 2.2 Animals

#### 2.2.1 ADHD Models

Sorokina et al. (2018) contained data from the striatum of mice selectively bred for a highly active, ADHD‐like phenotype. The original study used male mice from a selective breeding experiment (*n* = 12), developed for the testing of pharmacological or behavioral therapies in ADHD. Compared to the controls (*n* = 12), the ADHD‐like phenotype in the selectively bred mice was assessed with the Go/No‐Go test, the Y‐maze, Rotarod (an index of cerebellar function), and response to amphetamine treatment (Majdak et al. 2016). Half of the ADHD‐like mice (*n* = 6) and half of the controls (*n* = 6) were administered intraperitoneal amphetamine (10 mL/kg, 0.25 mg/kg d‐amphetamine sulfate in 9% saline) and the remaining half were administered the vehicle (10 mL/kg, 0.9% saline). The mice were sacrificed at 12 weeks old, and striatal samples were collected for RNA‐Seq.

The second RNA‐Seq dataset of an ADHD model, Nakano et al. (2023), contained rats exposed to methylphenidate paternally and displayed an ADHD‐like phenotype. At 6 weeks of age, the paternal rats were administered methylphenidate (15 mg/kg) or saline (10 mg/kg) subcutaneously for 21 days. In the offspring, a spontaneous locomotor activity test, the elevated plus maze, and a passive avoidance procedure confirmed that paternal exposure to the stimulant methylphenidate, often used to treat human ADHD, caused an ADHD‐like increase in locomotor activity, higher impulsivity, and impaired learned inhibition at 6–7 weeks of age (Nakano et al. 2023). Offspring from methylphenidate‐exposed (*n* = 3; 1 M, 2F) and saline‐exposed rats (*n* = 3; M2, F1) were sacrificed at 6 weeks, and samples of the striatum were collected for RNA‐Seq.

Yoshida et al.’s (2014) study was used for analysis as rodents were of the same sex as the other ADHD models (male) and of similar age (6 weeks), and there was no available expression data for SHRs exclusive to the striatum in NCBI GEO.

#### 2.2.2 ND Models

Several behavioral paradigms can test an animals’ preference for a drug, including conditioned place preference (CPP) and self‐administration experiments (Spanagel et al. 2017). Yang et al. (2017) used mice with green fluorescent protein in somatostatin‐positive interneurons (*n* = 6) and mice with yellow fluorescent protein in layer V pyramidal neurons (*n* = 6). Half of the animals in each group were administered nicotine (48 mg/kg/day) and half saline vehicle subcutaneously via an osmotic mini‐pump for 14 days. This dose of nicotine is known to alter brain activity, gene expression, and induce conditioned place preference in mice (Henley et al. 2013; Xiao et al. 2009).

The second ND model, Kozlova et al. (2021), had male rats self‐administer nicotine (30 μ g/kg/infusion) in six 2‐h sessions/day (10 infusions max). One group of rodents were offspring of nicotine‐sensitized rodents, and these offspring (*n* = 4) showed significantly increased self‐administration over the 6 days. Control rodents (*n* = 4) were given saline and showed no difference in self‐administration (Kozlova et al. 2021). Animals were sacrificed 3 days after the last self‐administration session, and samples of the NAc, nucleus accumbens shell (NAsh), and VTA were harvested. Only the NAc and NAsh samples were re‐analyzed in the present analysis. For all ND and ADHD models, mice were of the species *Mus musculus*, and rat species were *Rattus norvegicus*.

### 2.3 Sample Preparation

Raw sequence reads for each RNA‐Seq dataset were uploaded to Galaxy (Galaxy Community 2022; https://galaxyproject.org/) from NCBI GEO via SRA run selector. The resulting FASTQ files of paired cDNA reads were aligned by HISAT2 (Kim et al. 2019) to the reference genome, RGSC 6.0/rn6 for rats and GRCm39 for mice. HTSeq‐count (Anders et al. 2015) sorted and counted aligned reads into one BAM file per sample, and DESeq2 (Love et al. 2014) used this result to perform differential gene expression (DGE) analysis. Bioinformatics and Evolutionary Genomics (https://www.vandepeerlab.org/) created diagrams of DEGs shared between datasets. For SHRs, which uniquely contained microarray data, DGE analysis was conducted GEO2R (Love et al. 2014; https://www.ncbi.nlm.nih.gov/geo/geo2r/).

GEO2R uses limma (Smyth, 2005) for DGE analysis, which utilizes linear models and empirical Bayes methods (Ritchie et al. 2015). DESeq2 uses the same general statistical methods as limma but has a slightly different Bayesian calculation for determining dispersion (Love et al. 2014). Each produced tables with rows of genes and columns of the corresponding LogFC, *p*‐value, and FDR.

Sorokina et al. (2018) uniquely administered amphetamines to half of the rodents in the control and ADHD‐phenotype groups, so the effect of medication was assessed by performing DGE analysis on medicated rodents in the ADHD model group versus controls administered the vehicle. Other than Sorokina et al. (2018), each dataset was analyzed individually to obtain one list of DEGs with the associated expression statistics.

### 2.4 Enrichment Analysis in PathfindR

In RStudio (version 3.12.1; R Core Team 2021), the PathfindR package (https://cran.r‐project.org/web/packages/pathfindR/vignettes/intro_vignette.html) was used to cluster the genes and identify associated biological processes (Ulgen et al. 2019). PathfindR uses a protein– interaction network (PIN) to search for and rank active subnetworks, or groups of genes belonging to a PIN, from expression data. Next, PathfindR uses the active subnetworks to search known gene sets/pathways and identify enriched terms. In PathfindR, 10 iterations of subnetwork searches were run using the greedy search (GR) method, and FDR was used to set the enrichment threshold to 0.05 (Benjamini and Hochberg 1995). IntAct (del Toro et al. 2022; https://www.ebi.ac.uk/intact/) was selected as the PIN, and sets of 10–300 genes were downloaded from KEGG (Kanehisa et al. 2022; http://www.kegg.jp/), Reactome (Milacic et al. 2023; http://www.reactome.org), BioCarta (Rouillard et al. 2016; http://www.biocarta.com/), and GO‐All (Aleksander et al. 2023; http://www.geneontology.org/).

### 2.5 Network Analysis in Cytoscape

Gene members of pathways identified in multiple ND and ADHD datasets were explored further with network analysis. For a given pathway enriched in multiple datasets, the genes contributing to enrichment were searched in Cytoscape (Shannon et al. 2003; http://cytoscape.org) via STRING protein query (Szklarczyk et al. 2023; https://string‐db.org/), selecting for Mouse or Rat as the species as appropriate. Individual networks for each ADHD and ND dataset were constructed for every shared pathway identified in PathfindR. After constructing networks of shared pathways for each dataset, individual networks from ADHD models were merged for shared pathways as were individual ND networks.

### 2.6 Statistical Analysis

DEGs were considered features with a *p*‐value < 0.05, FDR < 0.05, and |LogFC| > 1.5. For the results from PathfindR, significant active subnetworks were selected according to default values (score_quan_thr = 0.8; sig_gene_thr = 0.02). The significant genes and pathways from each cohort were compared between rodent models of the same condition—ND or ADHD—and between ND and ADHD models. The results and discussion focus on DEGs and pathways that remained enriched after medication treatment in ADHD models.

## 3 Results

### 3.1 DEGs in ADHD and Nicotine Preference

Of the three ADHD models analyzed—HA, PM, and SHR–six DEGs were shared between PMs and SHRs, one between PMs and HAs, and one between HAs and SHRs (Table S1). The HA rodents treated with amphetamines from Sorokina et al. (2018) shared five DEGs with the HA rodents treated with the vehicle. For ND, Kozlova et al. (2021), with data from NAc and NAsh tissue, and Yang et al. (2017), with data from cortical pyramidal neurons (Thy1) and striatal interneurons (SST+), shared 10 DEGs between studies. The two cohorts from Yang et al. (2017) shared 11 DEGs and NAc and NAsh tissue from Kozlova et al. (2021) shared 14. Between samples and publications, most genes were similarly regulated (Table S2).

**FIGURE 1:**
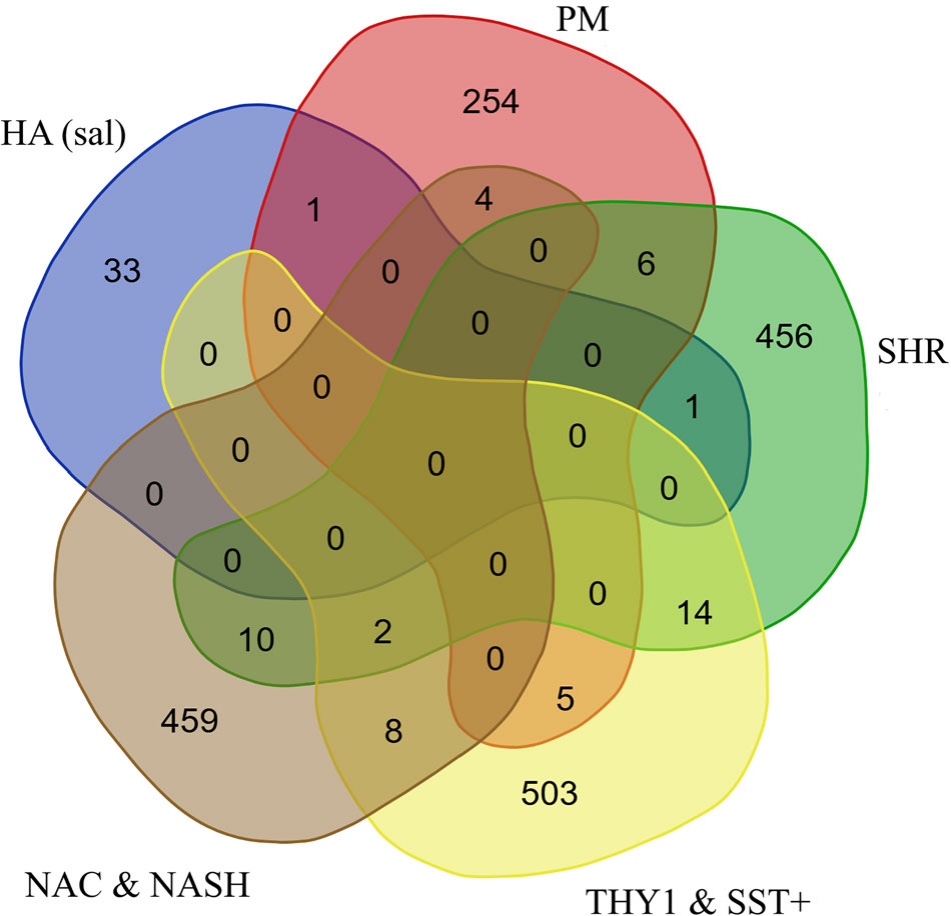
DEGs shared by at least one rodent model of ADHD and one of ND. Representation of the number of DEGs shared between ADHD and nicotine datasets. SHRs shared two DEGs with both Kozlova et al. (2021) (NAc and NAsh) and Yang et al. (2017) (Thy1 and SST+), 10 exclusively with Kozlova et al. (2021), and 14 exclusively with Yang et al. (2023). PMs shared four with Kozlova et al. (2021) and five with Yang et al. (2017).

Collectively, the three models of ADHD shared 16 DEGs with nicotine preference datasets from Kozlova et al. (2021) and 23 with Yang et al. (2017) (Figure 1, Table 1). Of these shared genes, many have been implicated in ADHD and/or ND: *ANK3*, *CALD1*, *CMTM8*, *ICAM2*, and *SYNPO*. Many cognitive deficits and neuropsychiatric disorders, including ADHD, are associated with mutations or SNPs in the *ANK3* gene (Iqbal et al. 2013). *ANK3* encodes AnkyrinG, which has three brain‐specific isoforms involved in action potential propagation and post‐synaptic glutamatergic signaling (Sobotzik et al. 2009). Analysis of gene imprinting in ADHD suggests *CALD1* contributes to its heritability (Smajlagic et al. 2019), and genomic analysis of pediatric ADHD patients reveals SNPs in the *CMTM8* gene (Pagerols et al. 2018), although it is unclear how these genes relate to the characteristics of ADHD.

**TABLE 1:** Names of DEGs shared between ADHD and ND datasets from Figure 1.

Cohorts	#DEGs	Gene IDs
SHR; NAsh; SST+	2	*CSF1*, *KIF13A*
SHR; NAc	4	*CALD1, DGUOK, HAPLN2, OLFML3*
SHR; NAsh	6	*AGAP3, ANK3, ARHGEF1, CMTM8, EIF4H, ITGA6*
SHR; SST+	9	*CAV2, CORO2A, GIPC2, NF2, SYNPO, TTC9, TUBE1, XRCC3, ZDBF2*
SHR; Thy1	5	*GNK1, ICAM1, LACC1, NT5DC1, RSPH10B*
PM; NAc; NAsh	1	*UBE2M*
PM; NAsh	3	*FLNA, IL34, PRKAR1B*
PM; SST+	2	*CCK, SATB2*
PM; Thy1	3	*LMO2, STK33, TRPM3*

*ICAM2* is an adhesion molecule known to play a role in synaptic plasticity. ADHD patients have higher *ICAM2* expression which is reduced by methylphenidate (Alaşehirli et al. 2015). The autism‐related gene *SYNPO* is associated with ADHD‐like behaviors and is predicted to be involved in intellectual disorders via its contributions to synapse maintenance and late synaptic plasticity (H. ‐T. Hu et al. 2023).

### 3.2 Pathway Analysis for ADHD and Nicotine Preference

PathfindR revealed KEGG, Reactome, BioCarta, and Gene Ontology terms significantly enriched in each ADHD and nicotine dataset. Between the ADHD models, pathways involving neurotrophic and TRK activity, RAS and mitogen‐activated protein kinase (MAPK) signaling, extracellular signal‐regulated kinase (ERK)/MAPK targets, postsynaptic signal transduction, and interleukin signaling were enriched in at least two of the three datasets. Pathways involving TRK activity, RAS/MAPK signaling, and ERK/MAPK targets remained upregulated in the striatum after amphetamine treatment in the one ADHD model for which this data was available.

For nicotine preference, the two datasets from Kozlova et al. (2021) and two from Yang et al. (2017) shared 10 pathways between all four, and 71 pathways between at least three of the datasets. FoxO signaling, NMDA receptor (NMDA‐R) pathways, long term potentiation, neurotrophin signaling, DA signaling, and two addiction pathways were shared between at least three. The cell lines from Yang et al. (2017) shared enrichment of NF‐kB signaling and interleukin‐1 family signaling with NAsh tissue from Kozlova et al. (2021; Table S3).

**FIGURE 2:**
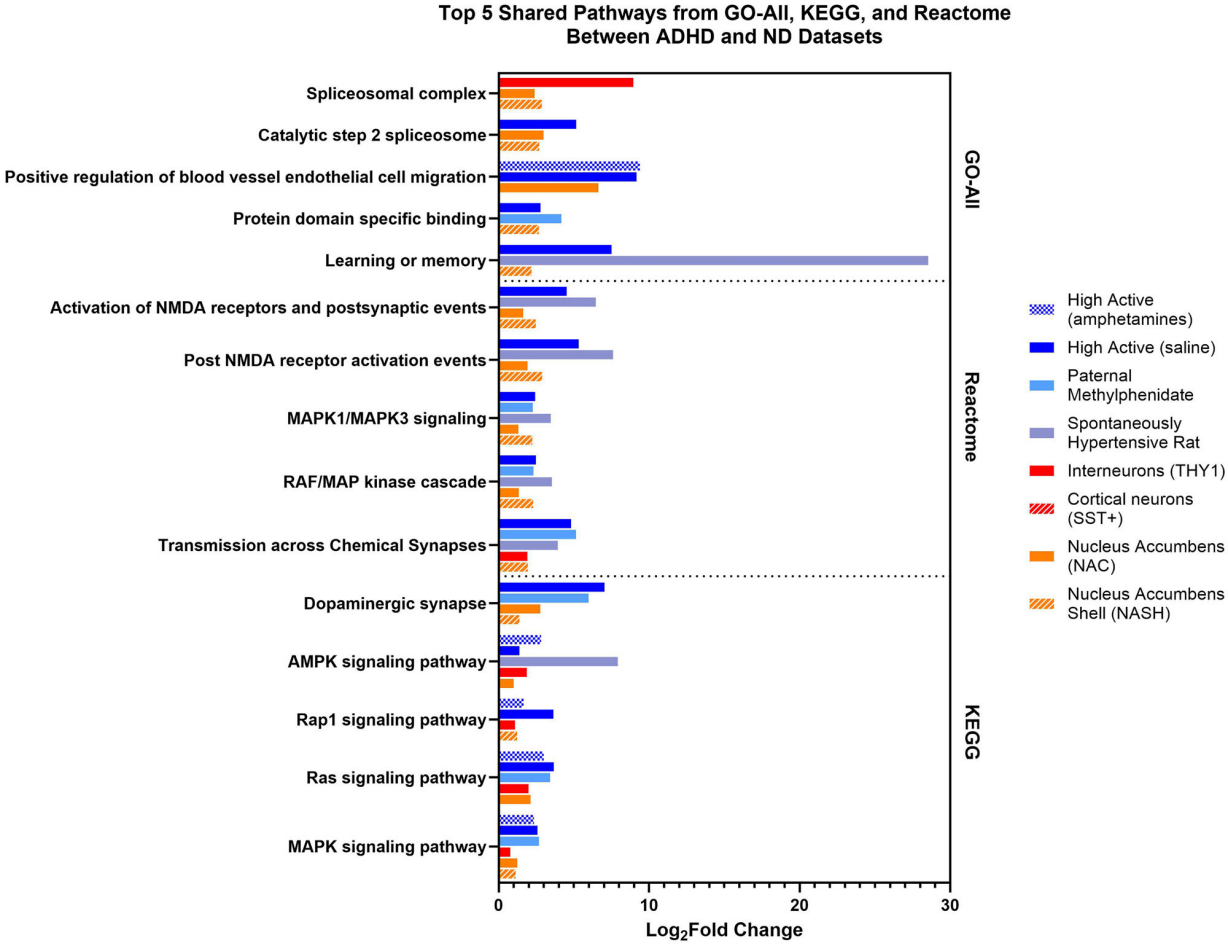
Top 5 enriched pathways from PathfindR shared between ND and ADHD models.

The top five enriched terms shared between ADHD and ND models across the pathway databases are outlined in Figure 2. Pathways related to ERK/MAPK, neurotransmitter, and synaptic transmission signaling were enriched in both ADHD and ND models.

### 3.3 Network Analysis for ADHD and Nicotine Preference

**FIGURE 3:**
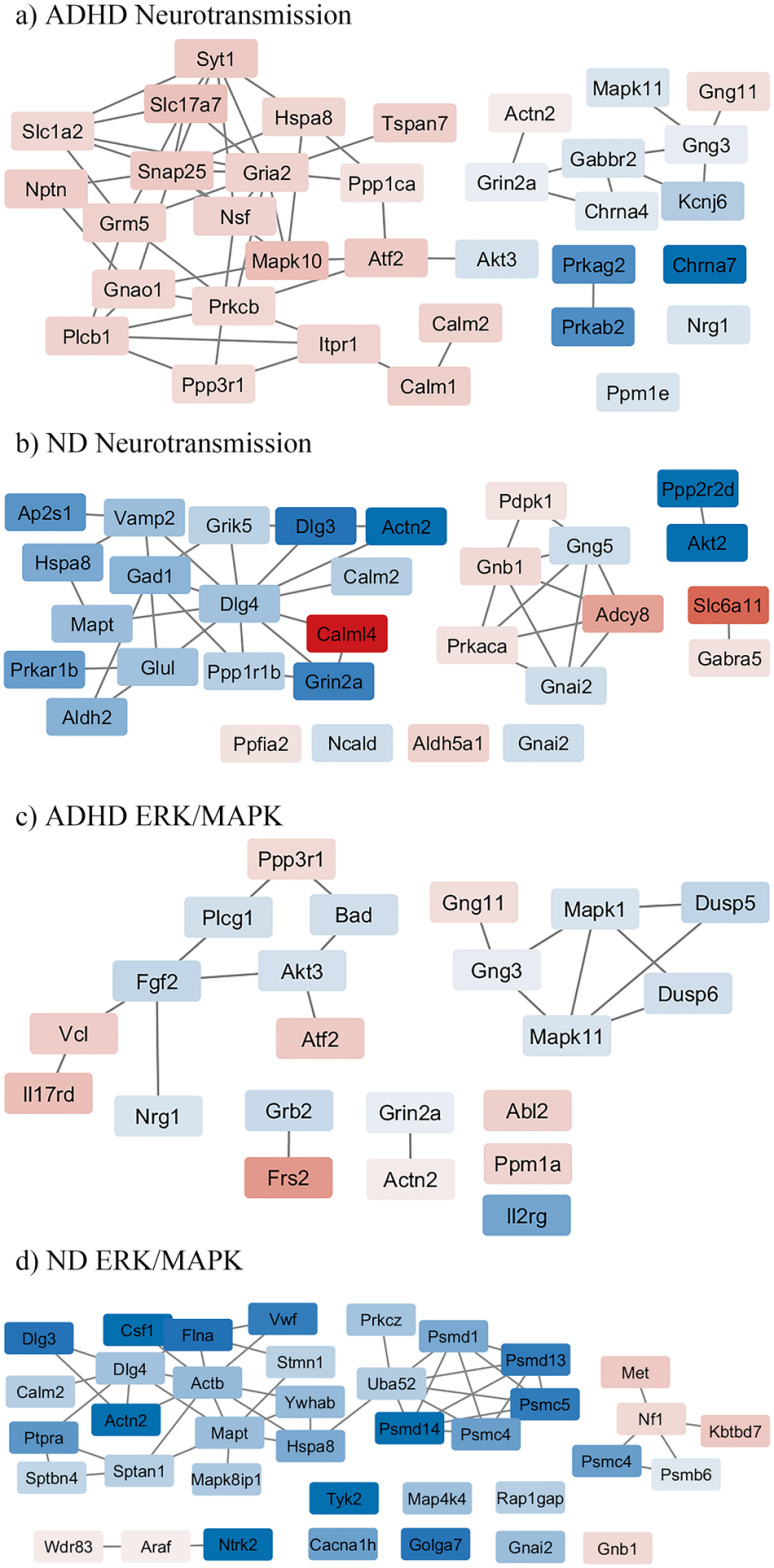
Gene networks of enriched neurotransmission pathways (hsa04728, R‐HAS‐112315, R‐HSA‐442755) and ERK/MAPK‐related signaling pathways (hsa04010, hsa04014, hsa04015, R‐HSA‐5673001, R‐HSA‐5684996) shared between rodent models of ADHD (a‐b) and ND (c‐d). Shading represents Log_2_FC (red = downregulated; blue = upregulated).

The pathways shared between ADHD and ND datasets are related mostly to neurotransmitter signaling and ERK/MAPK‐related signaling. The genes identified by PathfindR as contributors to the enrichment of these pathways in the ADHD and ND models were used to construct networks for neurotransmitter (Figure 3a,b) and ERK/MAPK signaling (Figure 3c,d).

PathfindR identified transmission across chemical synapses, dopaminergic synapses, and two NMDA‐R‐related pathways in at least two ADHD and two ND models, and the genes contributing to enrichment were mapped in Cytoscape. From these genes, Cytoscape identified enrichment of glutamatergic, GABAergic, dopaminergic, and cholinergic neurotransmission in the ADHD models. The downregulated gene cluster is involved in synapse maintenance, neurotransmission, and postsynaptic signal transduction. Together, they indicate a decrease in neuronal activity in the striatum, a key component of ADHD in humans.

Two cholinergic receptor subtypes (*CHRNA4*, *CHRNA7*) were upregulated in ADHD models, as was NMDA‐R subunit GluN2A, encoded by *GRIN2A*. *GRIN2A* was first associated with ADHD in 2002 (Smalley et al. 2002), and later research has replicated these findings (Turic et al. 2004; Adams et al. 2004; Williams et al. 2012). Cholinergic receptor alpha subunit genes *CHRNA4* and *CHRNA7* have been associated with both ADHD and nicotine use. In ADHD, nAChRs containing α4 and α7 subunits are candidate genes for their role in learning and attention via modulation of striatal DA release (Kessi et al. 2022). Chronic ND is shown to upregulate α4 and α7 nAChR subunits on GABA interneurons, which is known to modulate DA transmission (Sherafat et al. 2021).

Eight of the 12 genes contributing to the enrichment of the dopaminergic synapse pathway (hsa04728) were downregulated (*ATF2*, *GNAO1*, *GNG3*, *GRIA2*, *ITPR1*, *KCNJ6*, *MAPK10*, *MAPK11*, *PLCB1*, *PPP1CA*, *PPP3R1*, and *PRKCB*), and only *AKT3, GNG3*, *KCNJ6*, and *MAPK11* was upregulated. These genes are involved in signal transduction downstream of DA receptor activation and indicate a pattern of deficient DA signaling in the ADHD models. Other genes involved in neurotransmission downstream of receptor binding were also identified in ADHD models, including two upregulated AMPK genes, *PRKAB2* and *PRKAG2*. Via calcium/calmodulin‐dependent protein kinase 2 (CAMKK2), NMDA‐R‐mediated calcium influx can stimulate AMPK signaling (Mairet‐Coello et al. 2013). Further, *MAPK1* was slightly upregulated, and calcium influx from NMDA‐Rs is known to lead to ERK signaling.

Nicotine also resulted in differential expression of genes related to glutamate signaling, including glutamate‐inactivation enzyme *GLUL* (upregulated) and glutamate ionotropic receptor kainite type 5 *GRIK5* (upregulated). *GLRB* was downregulated and encodes the inhibitory glycine B receptor, and glycine is an NMDA‐R co‐agonist. Genes related to GABAergic transmission were identified in ND, including downregulated *SLC6A1* and *GABAR5*, and upregulated *GAD1*. *SLC6A1* encodes for the GABA reuptake transporter, *GAD1* for a GABA‐synthesis enzyme, and *GABAR5* for the alpha‐5 subunit of the GABA‐A receptor.

Two G protein‐coupled receptor (GPCR) gamma subunits were differentially expressed in ADHD models; *GNG3* was upregulated and *GNG11* was downregulated. GPCR subunits *GNG3* and *GNG11* have not been associated directly with ADHD but are implicated in cognitive ability (Ruano et al. 2010). Overall, ADHD is associated with deficits in some measures of cognitive ability including IQ, educational attainment, and reasoning skills (Demontis et al. 2019). Additionally, *KCNJ6* was upregulated, and this gene encodes for an inwardly rectifying potassium channel GIRK2. GRIK2 causes hyperpolarization in response to inhibitory GPCR signaling from D2 DA receptors and GABA‐B receptors in the striatum (Guatteo et al. 2004). Notably, GABA‐B receptor subunit *GABBR2* was slightly upregulated in ADHD models. GRIK2 is known to increase reward dependence when overexpressed (Ziegler et al. 2020), and SNPs in the *KCNJ6* gene have been associated with ND (Saccone et al. 2007).

ND rodents showed downregulation of two GPCR subunits (*GNB1*, *GNG5*) and upregulation of the GPCR subunit (*GNAI2*). neurocalcin delta (*NCALD)* was upregulated and regulates GPCR signal transduction and contributes to adult neurogenesis (Upadhyay et al. 2019). Additionally, several downstream signaling proteins were identified, including upregulated protein kinase A (PKA) subunits (*PRKACA*, *PRKAR1B*), downregulated adenylate cyclase 8 (*ADCY8*), and a downregulated protein tyrosine phosphatase receptor (*PTPRF*). GPCRs activate PKA, and PKA inhibits ADCY8 (Q. Wang et al. 2018).

Three genes related more generally to synaptic transmission were upregulated in ND—*AP2S1*, *VAMP2*, and *CALM1*. *AP2S1* encodes for a subunit of Adaptor Related Protein Complex 2 (CLAPS2), and *VAMP2* encodes for a vesicle‐associated membrane protein (VAMP‐2). CLAPS2 is involved in synaptic vesicle recycling (Gan and Watanabe 2018), and VAMP‐2 is a major soluble N‐ethylmaleimide sensitive factor attachment receptors (SNARE) protein involved in neurotransmitter release from vesicles into the synapse (Xi et al. 2020). The gene *CALM1* makes calmodulin 1, which acts in a calcium signaling pathway to control many ion channels, enzymes, and other signaling proteins (Solà et al. 2001).

In the ADHD models, *GRM5*, *SYT1*, *SNAP24*, and *TSPAN7* were downregulated, contributed to the enrichment of neurotransmission pathways, and are implicated in ADHD. A deletion in the metabotropic glutamate receptor gene *GRM5* was found in the affected parent and three offspring with ADHD (Elia et al. 2010), and knockout of *GRM5* in mice yields an ADHD‐like behavioral phenotype (Xu et al. 2021). Structural variances in *TSPAN7* are associated with intellectual disability, and the gene is relevant to ADHD given its role in D2‐like DA receptor trafficking (Lee et al. 2017). Synaptosomal‐Associated Protein 25 (SNAP‐25) is a component of SNARE complexes, which are essential for facilitating neurotransmitter release via vesicle fusion to synaptic membranes. SNARE complexes are regulated by proteins like synaptotagmin 1 (SYT1), which enables vesicle fusion (Davletov and Südhof 1994). Mice lacking *SNAP25* show hyperactive locomotion, abnormal catecholamine activity, and delayed behavioral development (Hess et al. 1992, 1996; Heyser et al. 1995). In ADHD, *SNAP25* is positively associated with severity (Sánchez‐Mora et al. 2013) and both *SNAP25* and *SYT1* are associated with ADHD susceptibility (Brookes et al. 2005, 2006; Forero et al. 2009; Guan et al. 2009; Gizer et al. 2009; Y. ‐S. Liu et al. 2017). A variant in *SYT1* (*SYT1*‐rs2251214) is associated with a history of social and behavioral deficits in ADHD patients (Cupertino et al. 2017).

Four of the genes found in synaptic transmission for ND have been previously associated with ADHD: *DLG4*, *GRIK5*, *PRKAR1B*, and *VAMP2*. Heritability in ADHD and other neurodevelopmental disorders has been associated with variations in the *PRKAR1B* gene (Marbach et al. 2021), and *GRIK5* is a predicted causal gene in ADHD (Fahira et al. 2019). *DLG4* and *VAMP2* may contribute to comorbid ADHD and epilepsy through their involvement in vesicle recycling and neurotransmission (Xi et al. 2020).

Several pathways related to RAS/RAF/MEK/ERK signaling and RAS/RAF independent ERK signaling were identified in both the ND and ADHD models. Generally, the RAS/RAF/MEK/ERK pathway is a signaling cascade in which ligand binding to a receptor tyrosine kinase activates RAS via a guanine nucleotide exchange factor that converts RAS to its active form, RAS‐GTP. RAS‐GTP activates RAF which in turn activates MEK, and MEK activates a MAPK called ERK. RAF, MEK, and ERK are activated via phosphorylation, and ERK enters the nucleus to induce gene expression. Alternatively, PKA and calcium‐dependent signaling events can bypass RAS and activate ERK.

Two of the upregulated genes associated with MAPK signaling in ADHD models, *DUSP5* and *DUSP6*, encode for dual specificity proteins (DUSP5 and DUSP6) and act as inhibitors of ERK signaling via negative feedback (Chen et al. 2019). DUSP6 specifically inactivates ERK1/2, coded by the *MAPK1* gene and slightly upregulated in ADHD. SNPs in *DUSP6* have previously been reported in ADHD and the DUSP6 protein is known to contribute to DA receptor trafficking (Demontis et al. 2019). Interleukin‐17 receptor D (*IL17RD*) was downregulated and acts as a feedback inhibitor of MAPK signaling and ERK activation. Genes for three receptors were involved in ERK enrichment; *FRS2* (fibroblast growth factor receptor substrate, FRS2) was downregulated, *GRB2* (growth factor receptor bound protein 2, GRB2) was upregulated, and *IL2RG* (interleukin 2 receptor subunit gamma, IL‐2Rγ) was upregulated. FRS2 binds tyrosine protein kinase A (TRKA) to stimulate ERK signaling and is downregulated by DUSP6 (Ekerot et al. 2008). IL‐2Rγ induces ERK signaling through the JAK/STAT pathway and GBR2 by increasing RAS‐GTP concentrations (Lin & Leonard, 2018).

*DUSP8* was slightly downregulated in ND rodents, and the dual specificity protein it encodes (DUSP8) inhibits ERK (Martell et al. 1995). *GFRA1*, *NF1*, and *KBTBD7* were all downregulated in ND. *GFRA1* is a positive regulator of RAF/ERK signaling (Ibáñez et al. 2020), while *NF1* and *KBTBD7* are negative regulators (Anastasaki et al. 2022; Genau et al. 2015). *GFRA1* signaling is regulated by PTPRA phosphatase (Yadav et al. 2020), encoded by *PTPRA*, and upregulated in ND. Additionally, several proteasome 26S subunits were upregulated *PSMB6*, *PSMC4*, *PSMC5*, *PSMD1*, *PSMD12*, *PSMD13*, *PSMD14*) as was a ubiquitin protein (*UBB*). Proteasome 26S and associated ubiquitin proteins contribute to synaptic plasticity induced by ERK signaling (Hegde 2004). ND upregulated several genes encoding for proteins involved in downstream ERK‐signaling, including *PDE6G* and *TYK2*. *PDE6G* has been shown to induce ERK signaling (Wan et al. 2001). *TKY2* encodes tyrosine kinase 2 (JTK1) and induces the expression of growth factor‐responsive genes via interactions with JAK/STAT family members, activated by ERK signaling (X. Hu et al. 2021).

## 4 Discussion

### 4.1 Overview of Relevant DEG and Pathway Findings

The molecular signature of the striatum from rodent models of ADHD and ND reveals new candidate genes and pathways common to both disorders. This analysis explored differentially expressed genes (DEGs) and enriched pathways in the striatum of three models of ADHD (SHR, PM, HA) and four types of brain tissue from two studies of ND (NAc, NAsh, Thy1, SST+). Several genes identified in DGE and pathway analysis are implicated in human ADHD and ND (*ANK3*, *CALD1*, *CHRNA4*, *CHRNA7*, *CMTM8*, *DLG4*, *DUSP6*, *GNG3*, *GNG11*, *GRIK5*, *GRINA2, GRM5*, *ICAM2*, *KCNJ6*, *MAPK1*, *PRKAB1*, *PRKAG2*, *SNAP25*, *SYNPO*, *SYT1, VAMP2*). The rodent models of each disorder also shared several DEGs. Enrichment in RAS/RAF/MAPK, Rap1, and MAPK1/MAPK3 signaling, NMDA‐R events, and synaptic transmission pathways were identified by pathway analysis in both ND and ADHD models.

This analysis suggests the MAPK/ERK signaling pathway is upregulated in ADHD and ND models. Several of the pathways related to ERK/MAPK enrichment remained enriched in the ADHD model treated with amphetamines from Sorokina et al. (2018). The enrichment of ERK/MAPK in the striatum does not appear to be suppressed by medication. This pathway may be relevant to increased ND in ADHD patients, as the association between ADHD and ND is similarly unaffected by pharmacological treatment for ADHD in humans.

### 4.2 Neurotransmission Is Dysregulated in ADHD and ND Models

While no genes for monoamine receptors, transporters, or synthesis enzymes contributed to enriched neurotransmission in the ADHD models, the gene for the inwardly rectifying potassium channel GRIK2 (*KCNJ6*) activated by D2 DA receptors was upregulated, and eight genes downstream of DA receptors were downregulated. In ND models, upregulation of PKA subunits (*PRKACA*, *PRKAR1B*) suggests increased excitatory dopaminergic activity in the striatum as PKA acts downstream of D1‐Rs. The genes involved with enrichment of the DA synapse mostly contribute to signaling cascades downstream of DA receptors in both ND and ADHD.

Genes related to glutamatergic signaling and NMDA‐R‐mediated glutamate signaling were identified in all three ADHD models including upregulated AMPK subunits (*PRKAB2*, *PRKAG2*) and NMDA‐R subunit (*GRIN2A*). NMDA‐R activation leads to calcium influx, activating CaMKK2, which induces AMPK signaling. Some researchers suggest that the irregular timing of glutamate and DA firing in the striatum contributes to the differences in striatal morphology and activation observed in ADHD patients (Moore et al. 2006; Kollins & Adcock, 2014). SHRs also show increased striatal glutamate signaling (Miller et al. 2014; Cheng et al. 2017).

Pathways related to NMDA‐R activity were also enriched in three of the four ND datasets. NMDA‐R activity contributes to nicotine addiction by inducing long‐term potentiation (LTP) in the striatum and PFC. Nicotine's agonism of α4‐containing nAChRs on cell bodies and α7 nAChRs on presynaptic terminals of glutamate neurons initially increases their innervation of DA neurons. The α4‐containing nAChRs are quickly desensitized reducing glutamatergic firing (Couey et al. 2007; Licheri et al. 2018), but if the initial increase leads to NMDA‐R activation on DA neurons, LTP can occur (Mansvelder and McGehee, 2000). This LTP is required for learning of nicotine cues that leads to craving and anticipation of nicotine reward and is thought to be dependent on NMDA‐Rs—selective knockout of NMDA‐Rs on DA neurons of the VTA prevents nicotine CPP and is facilitated by α4 and α7 containing nAChRs (L. P. Wang et al. 2010; Ávila‐Ruiz et al. 2014). These same subunits (*CHRNA4, CHRNA7*) were upregulated in the striatum of the ADHD models.

### 4.3 ERK Signaling Is Dysregulated in Rodent Models of ADHD and ND

Several enriched ERK/MAPK‐related pathways in the ADHD models (i.e., Rap1 signaling, Ras signaling, MAPK signaling, MAPK1/MAPK3 signaling, RAF/MAPK signaling) indicate increased ERK signaling in the striatum. Network analysis revealed the upregulation of two genes and downregulation of one that inhibits ERK signaling (*DUSP5*, *DUSP6*, and *IL17RD*), two genes that transduce signals upstream of ERK (*GRB2*, *IL2RG*), and the gene that encodes the ERK2 protein (*MAPK1*). This expression pattern in the ADHD models is consistent with enhanced and exhaustive ERK signaling (Arkell et al. 2008; Seo et al. 2017; Kung and Sudhamsu 2023). There is limited research on ERK2 signaling in ADHD, but some evidence warrants the investigation of ERK/MAPK signaling in the disorder. Disorders RASopathies, a family of syndromes characterized by excessive ERK/MAPK signaling from mutations to ERK or upstream regulators, result in similar behavioral issues to those observed in ADHD patients (Green et al. 2017; Montanaro et al. 2022). RASopathies are highly co‐occurring with ADHD, altogether suggesting the two disorders may be pathologically linked (Alfieri et al. 2014; Iroegbu et al.^2021^). Alternatively, ERK1 knockout in mice increases locomotor activity, a common characteristic of ADHD models. However, these mice are hypersensitive to amphetamines suggesting the hyperactivity is inconsistent with ADHD (Engel et al. 2009). Overexpression of ERK has not been explored, but upstream of ERK overactive MEK1 signaling in GABAergic neurons is linked to behavioral inhibition deficits in mice (Holter et al. 2021).

The same ERK/MAPK‐related pathways were upregulated in ND models. The ERK/MAPK pathway has previously been implicated in ND. ERK1/2 phosphorylation contributes to the rewarding effects of nicotine (Morella et al. 2022), and regulation of neuroplasticity genes in cortical neurons is required for nicotine‐induced CPP in mice (Fan et al. 2023). Steiner et al. (2007) showed that higher rates of ERK phosphorylation from nicotine increase self‐administration in mice. Importantly, ERK1/2 phosphorylation in the striatum from nicotine results from synchronous D1‐R and NMDA‐R activation and contributes to the rewarding effects of nicotine. It also induces long‐term synaptic changes in the striatum that are required for drug dependence (Valjent et al. 2005). Therefore, ERK activation contributes to the long‐term association of nicotine with contextual cues throughout the brain, short‐term nicotine reward in the striatum, and neuroadaptations to striatal reward circuitry necessary for drug dependence explaining the enrichment of these pathways in the ND models. Activation of D1‐Rs by nicotine increases this effect, as PKA induces a signaling cascade that activates ERK1/2 (see Cahill et al. 2014). NMDARs can also activate PKA, inducing synaptic plasticity through ERK/MAPK signaling (Waltereit and Weller 2003; Hetman and Kharebava 2008). Knockout of D1‐Rs or antagonism of NMDA‐Rs blocks striatal ERK2 phosphorylation, suggesting the involvement of both receptors in this pathway (Valjent et al. 2000, 2005).

Overall, the striatum of the ADHD models may exacerbate nicotine‐induced gene expression changes in three distinct ways. First, the nAChR subunits (α4 and α7) known to facilitate synchronized NMDA‐R and D1‐R signaling were upregulated in ADHD models. Second, an NMDA‐R subunit was upregulated in ADHD models suggesting a greater likelihood of a synchronized firing event. Third, EKR2 phosphorylation from converging NMDA‐R and D1‐R firing could occur more quickly in ADHD models as ERK/MAPK signaling was enriched and EKR2 mRNA was slightly upregulated. Therefore, the striatum of the ADHD models may accelerate this nicotine‐induced pathway, suggesting a mechanism for the more rapid development and higher occurrence of ND in humans with ADHD (e.g., Molina et al. 2018).

### 4.4 Directions for Future Research

**FIGURE 4:**
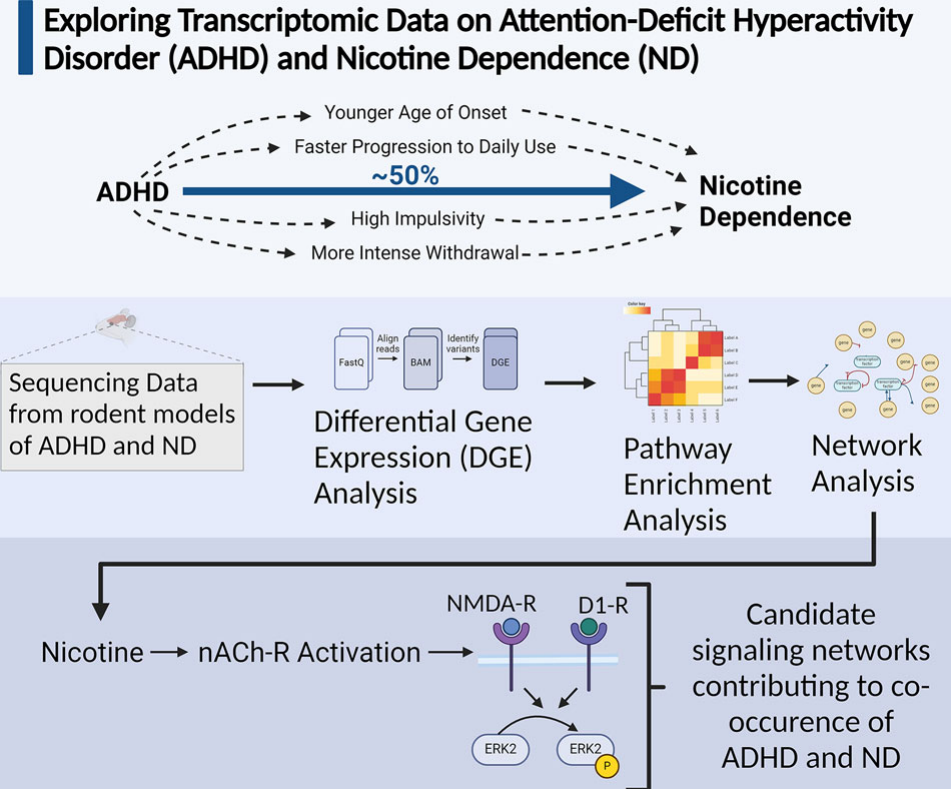
Graphical abstract of the experiment outlining rationale, methods, and key results (created with BioRender.com).

The literature supports physiological and behavioral dysfunction in patients with ADHD related to impulsivity, emotional regulation, and inhibitory control. To compensate for the executive and cognitive impairments characteristic of ADHD, some patients are prescribed stimulant medications, but many self‐medicate instead of, or in addition, to these drugs. Almost half of ADHD patients are nicotine‐dependent (Pomerleau et al. 1995; Mitchell et al. 2018), and lung cancers are more prevalent in ADHD than in the general population (Demontis et al. 2019). The present analysis used public RNA‐Seq data from the striatum and whole brain of three rodent models of ADHD and two of ND to explore gene expression patterns and biological pathway enrichment to identify candidate mechanisms that may relate to the high co‐occurrence between the two disorders in humans (Figure 4). We identified several DEGs in rodent models of ADHD and ND that have previously been implicated in each disorder, and pathway analysis revealed enrichment of several pathways related to synaptic transmission and ERK/MAPK signaling. The method was cost‐effective, as it did not require the use of animals, and the analysis was conducted in open‐source software.

While these results do not predict in vivo patterns of neuronal excitability or provide direct mechanistic evidence linking ND and ADHD, they reveal a transcriptional pattern in the striatum that may play a causal role in the development and persistence of ND in ADHD. Although ERK/MAPK‐related signaling has been implicated in both ND and ADHD to some extent, further research is needed to determine if these pathways are dysfunctional in human ADHD, associated with ADHD‐like behavioral characteristics in humans, and relevant to ND. Importantly, these pathways remained enriched after treatment with amphetamines in one ADHD model, indicating that ADHD medications may not prevent ND if potentiated by this mechanism. As current treatments for ADHD do not appear to change the likelihood of ND in these patients, understanding the role of D1‐R/NMDA‐R mediated ERK signaling in these patients may open new avenues for future treatment.

## Author Contributions

**Sarah Van Horn**: investigation, conceptualization, writing–original draft, funding acquisition, formal analysis. **Heather Driscoll**: supervision, resources, writing–review and editing, methodology. **Donna J Toufexis**: writing–review and editing, supervision, resources, methodology.

## Ethics Statement

Ethical review is not required as this study analyzed anonymized data in the public domain.

## Conflicts of Interest

The authors declare no conflicts of interest.

### Peer Review

The peer review history for this article is available at https://publons.com/publon/10.1002/brb3.70444.

## Supporting information



Supporting Information

## Data Availability

Supplemental datasets, including DGE and PEA results, are available upon request. The data that support the findings of this study are available in NCBI Gene Expression Omnibus at https://www.ncbi.nlm.nih.gov/geo/. These data were derived from the following resources available in the public domain: ‐ GSE116752, https://www.ncbi.nlm.nih.gov/geo/query/acc.cgi?acc=GSE116752‐GSE211982, https://www.ncbi.nlm.nih.gov/geo/query/acc.cgi?acc=GSE211982‐GSE41452, https://www.ncbi.nlm.nih.gov/geo/query/acc.cgi?acc=GSE41452‐GSE89899, https://www.ncbi.nlm.nih.gov/geo/query/acc.cgi?acc=GSE89899‐GSE157683, https://www.ncbi.nlm.nih.gov/geo/query/acc.cgi?acc=GSE157683.
