# Supplementary material for: Transcriptomic Dysregulation in Animal Models of Attention‐Deficit Hyperactivity Disorder and Nicotine Dependence Suggests Shared Neural Mechanisms
Source: Brain Behav. 2025 Mar 26;15(3):e70444. doi: 10.1002/brb3.70444 (PMC11938115; doi:10.1002/brb3.70444)
Supplement: Supplementary file 1 — Supporting Information [file BRB3-15-e70444-s001.docx]

**Supplemental Materials**

*Supplemental Table 1:*

| GeneID | HA (amp) | | HA (sal) | | PM | | SHR | |
| --- | --- | --- | --- | --- | --- | --- | --- | --- |
|  | **LogFC** | **pval** | **LogFC** | **pval** | **LogFC** | **pval** | **LogFC** | **pval** |
| *Gm31373* | 2.763 | <0.001 | 2.752 | <0.001 |  |  |  |  |
| *Gm54152* | 1.762 | <0.001 | 2.481 | <0.001 |  |  |  |  |
| *Nop56* | 2.004 | <0.001 | 2.303 | <0.001 |  |  |  |  |
| *Doc2g* | 1.634 | <0.001 | 3.457 | <0.001 |  |  |  |  |
| *Trmt10b* | -2.907 | <0.001 | -2.877 | <0.001 |  |  |  |  |
| *Dnah8* |  |  | 1.509 | <0.001 | 2.518 | <0.001 |  |  |
| *Cx3cr1* |  |  | -1.5991 | <0.001 |  |  | 1.8294 | 0.002 |
| *Mfsd11* |  |  |  |  | 3.863 | 0.048 | 2.021 | 0.001 |
| *Dlc1* |  |  |  |  | 2.426 | 0.043 | -1.764 | 0.001 |
| *Sec1* |  |  |  |  | 1.841 | 0.040 | 2.874 | 0.002 |
| *Pak3* |  |  |  |  | 1.747 | 0.005 | -1.992 | <0.001 |
| *Eif1a* |  |  |  |  | 2.552 | 0.030 | -3.290 | <0.001 |
| *Xlr4a* |  |  |  |  | 3.250 | 0.015 | 1.626 | 0.001 |

*Supplemental Table 1:* Shared DEGs between rodent models of ADHD. Log fold change (LogFC) and p-value (pval) are provided according to dataset for each shared feature. HA (amp) and HA (sal) correspond to the amphetamine and saline treated rodents in Sorokina et al. (2019, GSE116752), respectively. PM corresponds to the paternal methylphetnidate ADHD model from Nakano et al. (2023, GSE211982), and SHRs are spontaneously hypertensive rats from Yoshida et al. (2014, GSE41452).

*Supplemental Table 2:*

| **GeneID** | **NAC** | | **NASH** | | **Thy1** | | **SST+** | |
| --- | --- | --- | --- | --- | --- | --- | --- | --- |
|  | **LogFC** | **pval** | **LogFC** | **pval** | **LogFC** | **pval** | **LogFC** | **pval** |
| *Pdp2* |  |  |  |  | -2.420730 | 0.042987 | 3.338881 | 0.009832 |
| *Sarm1* |  |  |  |  | 2.111405 | 0.017801 | -1.845468 | 0.030381 |
| *Sdad1* |  |  |  |  | 1.649952 | 0.026660 | 1.625073 | 0.015218 |
| *Adamts18* |  |  |  |  | -1.706969 | 0.042646 | 3.041956 | 0.009166 |
| *Pmp22* |  |  |  |  | 4.430151 | 0.040220 | 4.937592 | 0.024196 |
| *Zfp830* |  |  |  |  | 1.625787 | 0.026885 | -2.049645 | 0.041922 |
| *Gstm6* |  |  |  |  | 1.595802 | 0.020965 | 6.630842 | 0.003594 |
| *Elfn2* |  |  |  |  | -3.265432 | 0.034968 | -3.035849 | 0.013661 |
| *Magt1* |  |  |  |  | -1.712458 | 0.040661 | 1.859119 | 0.023239 |
| *Zfp708* |  |  |  |  | -5.135497 | 0.020938 | 6.063252 | 0.010963 |
| *Plk4* |  |  |  |  | -1.826162 | 0.045809 | -2.132174 | 0.016597 |
| *Amt* |  |  | 4.088529 | 0.030155 | -2.149662 | 0.013374 |  |  |
| *Nfrkb* |  |  | 4.305203 | 0.011820 | 1.559140 | 0.013653 |  |  |
| *Wfs1* |  |  | 2.686130 | 0.018133 | 1.734318 | 0.012941 |  |  |
| *Nell2* |  |  | 2.549436 | 0.004724 |  |  | 6.506929 | 0.002087 |
| *Csf1* |  |  | 4.193460 | 0.022863 |  |  | -2.489576 | 0.033475 |
| *Kif13a* |  |  | 4.801661 | 0.002134 |  |  | -2.692829 | 0.027531 |
| *Ptk2b* |  |  | 1.775725 | 0.028022 |  |  | 4.227629 | 0.032790 |
| *Plpp3* |  |  | 3.133266 | 0.013331 |  |  | 2.164576 | 0.014611 |
| *Anxa6* | 5.001307 | 0.002358 | 4.342207 | 0.002588 |  |  |  |  |
| *Apbb1* | 3.527682 | 0.035331 | 2.235540 | 0.030930 |  |  |  |  |
| *Apln* | 2.617822 | 0.008901 | -2.580364 | 0.041696 |  |  |  |  |
| *Atp5f1b* | 1.966721 | 0.023468 | 2.088489 | 0.001334 |  |  |  |  |
| *Kcnt1* | 3.131810 | 0.029139 | 3.131810 | 0.029139 |  |  |  |  |
| *Ndufv2* | 2.113409 | 0.036065 | 2.113409 | 0.036065 |  |  |  |  |
| *Nxf1* | 2.723441 | 0.023381 | 4.584582 | 0.012135 |  |  |  |  |
| *Phactr3* | 2.306516 | 0.004291 | 2.153086 | 0.011864 |  |  |  |  |
| *Rpl11* | 1.998366 | 0.019249 | 2.701221 | 0.047556 |  |  |  |  |
| *Rpl6* | 2.296218 | 0.014633 | 3.734013 | 0.010919 |  |  |  |  |
| *Rpl9-ps30* | 2.013965 | 0.030541 | 3.019684 | 0.034448 |  |  |  |  |
| *Sf3b1* | 2.836162 | 0.003054 | 3.394069 | 0.015738 |  |  |  |  |
| *Tubb4b* | 2.280302 | 0.018127 | 3.770084 | 0.030077 |  |  |  |  |
| *Ube2m* | 1.532321 | 0.028699 | 1.650471 | 0.027337 |  |  |  |  |
| *Ndrg1* | 4.012304 | 0.046714 |  |  |  |  | 4.594316 | 0.016757 |
| *Pus3* | 4.651832 | 0.004731 |  |  |  |  | 2.970778 | 0.036938 |

*Supplemental Table 2:* Shared DEGs between rodent models of ND. Log fold change (LogFC) and p-value (pval) are provided according to dataset for each shared feature. NAC = Nucleus Accumbens, NASH = Nucleus Accumbens shell (Kozlova et al. 2021, GSE157683). Thy1 = cortical pyramidal neurons, SST+ = striatal interneurons (Yang et al. 2017, GSE89899).

*Supplemental Table 3:*

| **Path-way ID** | **Description** | **HA (amp)** | | **HA (sal)** | | **PM** | | **SHR** | | **NAC** | | **NASH** | | **SST+** | | **THY1** | |
| --- | --- | --- | --- | --- | --- | --- | --- | --- | --- | --- | --- | --- | --- | --- | --- | --- | --- |
|  |  | **Fold Enrich** | **Supp.** | **Fold Enrich** | **Supp.** | **Fold Enrich** | **Supp.** | **Fold Enrich** | **Supp.** | **Fold Enrich** | **Supp.** | **Fold Enrich** | **Supp.** | **Fold Enrich** | **Supp.** | **Fold Enrich** | **Supp.** |
| R-HSA-5673001 | RAF/MAP kinase cascade |  |  | 2.4802 | 0.0578 | 2.3198 | 0.011 | 3.541 | 0.047 | 1.3455 | 0.005 | 2.3058 | 0.0431 |  |  | 1.107 | 0.011 |
| R-HSA-5684996 | MAPK1/MAPK3 signaling |  |  | 2.4255 | 0.0578 | 2.269 | 0.011 | 3.463 | 0.047 | 1.3158 | 0.005 | 2.2549 | 0.0431 |  |  | 1.203 | 0.011 |
| R-HSA-112315 | Transmission across Chemical Synapses |  |  | 4.8105 | 0.1294 | 5.142 | 0.011 | 3.924 | 0.016 |  |  | 1.9543 | 0.0122 |  |  | 1.908 | 0.011 |
| R-HSA-1169091 | Activation of NF-kappaB in B cells |  |  |  |  |  |  |  |  |  |  | 3.7694 | 0.08 | 0.604 | 0.008 | 0.977 | 0.032 |
| R-HSA-2871837 | FCERI mediated NF-kB activation |  |  |  |  |  |  |  |  |  |  | 2.3384 | 0.0649 | 1.125 | 0.008 | 0.909 | 0.021 |
| R-HSA-5607761 | Dectin-1 mediated noncanonical NF-kB signaling |  |  |  |  |  |  |  |  |  |  | 4.6553 | 0.0862 | 0.653 | 0.008 | 1.055 | 0.032 |
| R-HSA-5676590 | NIK-->noncanonical NF-kB signaling |  |  |  |  |  |  |  |  |  |  | 4.892 | 0.08 | 0.686 | 0.008 | 1.109 | 0.032 |
| R-HSA-446652 | Interleukin-1 family signaling |  |  |  |  |  |  |  |  |  |  | 2.1504 | 0.0671 | 0.536 | 0.008 | 1.3 | 0.016 |
| R-HSA-9020702 | Interleukin-1 signaling |  |  |  |  |  |  |  |  |  |  | 2.8735 | 0.0677 | 0.717 | 0.008 | 1.158 | 0.021 |
| R-HSA-442755 | Activation of NMDA receptors and postsynaptic events |  |  | 4.5187 | 0.0442 |  |  | 6.4510 | 0.0156 | 1.634 | 0.005 | 2.4711 | 0.0122 |  |  | 2.241 | 0.005 |
| R-HSA-438064 | Post NMDA receptor activation events |  |  | 5.3204 | 0.0442 |  |  | 7.5955 | 0.0156 | 1.924 | 0.005 | 2.9096 | 0.0122 |  |  | 2.111 | 0.011 |
| R-HSA-451927 | Interleukin-2 family signaling |  |  | 3.0811 | 0.006 |  |  | 10.703 | 0.016 |  |  |  |  |  |  |  |  |
| R-HSA-512988 | Interleukin-3, Interleukin-5 and GM-CSF signaling |  |  | 3.0811 | 0.006 |  |  | 10.703 | 0.016 |  |  |  |  |  |  |  |  |
| R-HSA-912526 | Interleukin receptor SHC signaling |  |  | 5.021 | 0.006 |  |  | 17.442 | 0.016 |  |  |  |  |  |  |  |  |
| R-HSA-112314 | Neurotransmitter receptors and postsynaptic signal transmission |  |  | 4.2411 | 0.0343 | 7.052 | 0.011 | 5.382 | 0.016 |  |  |  |  |  |  |  |  |
| R-HSA-373752 | Netrin-1 signaling |  |  | 5.7689 | 0.006 | 13.129 | 0.011 |  |  |  |  |  |  |  |  |  |  |
| hsa04010 | MAPK signaling pathway | 2.3447 | 0.0208 | 2.5770 | 0.0871 | 2.6783 | 0.021 |  |  | 1.2427 | 0.015 | 1.1275 | 0.0122 |  |  | 0.7767 | 0.005 |
| hsa04015 | Rap1 signaling pathway | 1.6551 | 0.0218 | 3.6382 | 0.1413 |  |  |  |  |  |  | 1.2380 | 0.0061 |  |  | 1.0965 | 0.01 |
| hsa04014 | Ras signaling pathway | 3.0013 | 0.0417 | 3.6651 | 0.1569 |  |  |  |  | 3.4282 | 0.005 |  |  |  |  | 1.9883 | 0.005 |
| hsa04724 | Glutamatergic synapse |  |  | 7.9294 | 0.0641 | 4.4500 | 0.011 |  |  | 1.1471 | 0.005 |  |  |  |  |  |  |
| hsa05030 | Cocaine addiction |  |  | 3.5092 | 0.0114 |  |  |  |  | 5.077 | 0.005 | 3.0705 | 0.0061 |  |  | 2.088 | 0.021 |
| hsa04720 | Long-term potentiation |  |  |  |  |  |  |  |  | 5.423 | 0.015 |  |  |  |  | 1.487 | 0.005 |
| hsa04020 | Calcium signaling pathway | 1.4554 | 0.0218 | 4.6209 | 0.1262 |  |  |  |  |  |  |  |  |  |  | 0.9642 | 0.005 |
| hsa04722 | Neurotrophin signaling pathway |  |  | 4.8920 | 0.1569 | 1.3074 | 0.010 |  |  | 2.022 | 0.005 |  |  |  |  | 1.109 | 0.016 |
| hsa04728 | Dopaminergic synapse |  |  |  |  | 5.9794 | 0.021 |  |  | 2.774 | 0.005 |  |  |  |  | 2.029 | 0.059 |
| hsa05031 | Amphetamine addiction |  |  | 8.7463 | 0.1413 |  |  |  |  | 7.23 | 0.005 |  |  |  |  | 1.983 | 0.016 |
| hsa04068 | FoxO signaling pathway |  |  | 2.1349 | 0.028 |  |  |  |  | 1.879 | 0.026 |  |  |  |  | 2.061 | 0.043 |
| hsa04550 | Signaling pathways regulating pluripotency of stem cells |  |  | 1.923 | 0.006 | 4.376 | 0.011 |  |  |  |  |  |  |  |  |  |  |
| hsa04152 | AMPK signaling pathway | 2.8373 | 0.0218 | 2.2785 | 0.006 |  |  | 7.9146 | 0.0156 | 1.0025 | 0.010 |  |  |  |  | 1.3746 | 0.005 |
| hsa04657 | IL-17 signaling pathway |  |  | 1.5063 | 0.006 |  |  |  |  |  |  |  |  |  |  | 0.3635 | 0.005 |
| M287 | Biocarta ERK Pathway | 0 | 0.0208 | 3.0543 | 0.011 | 0 | 0.011 |  |  | 0 | 0.005 |  |  |  |  | 0 | 0.005 |
| M17294 | Biocarta RAS Pathway | 0 | 0.0208 | 3.7484 | 0.011 | 0 | 0.011 |  |  | 5.4227 | 0.010 |  |  |  |  | 0 | 0.005 |
| M9070 | Biocarta CREB Pathway | 0 | 0.0208 | 7.4969 | 0.023 | 0 | 0.011 |  |  |  |  | 1.6399 | 0.006 |  |  | 0 | 0.005 |
| M9664 | Biocarta GPCR Pathway | 0 | 0.0208 | 11.375 | 0.127 | 0 | 0.011 | 0 | 0.016 | 0 | 0.005 | 2.4881 | 0.006 |  |  | 0 | 0.005 |

*Supplemental Table 3*: Pathways shared between three rodent models of ADHD and/or at least two rodent models of ND.

*Supplemental Table 4:*

a)

| **Network** | **Gene ID** | **HA** | | **PM** | | **SHR** | |
| --- | --- | --- | --- | --- | --- | --- | --- |
|  |  | **Log_FC** | **Padj** | **Log_FC** | **Padj** | **Log_FC** | **Padj** |
| **ADHD Neuro-transmission** | *ACTN2* |  |  | -0.326 | 0.017 |  |  |
|  | *AKT3* | 0.647 | 0.019 |  |  |  |  |
|  | *ATF2* | -1.198 | 0.045 |  |  |  |  |
|  | *CALM1* | -1.089 | 0.004 |  |  |  |  |
|  | *CALM2* | -0.996 | 0.006 |  |  |  |  |
|  | *CHRNA4* |  |  | 0.411 | 0.031 |  |  |
|  | *CHRNA7* |  |  |  |  | 4.291 | 0.043 |
|  | *GABBR2* |  |  | 0.589 | <0.001 |  |  |
|  | *GNOA1* | -0.895 | 0.029 |  |  |  |  |
|  | *GNG11* |  |  | -0.679 | 0.005 |  |  |
|  | *GNG3* |  |  | 0.317 | 0.035 |  |  |
|  | *GRIA2* | -0.991 | 0.038 |  |  |  |  |
|  | *GRIN2A* |  |  | 0.306 | 0.036 |  |  |
|  | *GRM5* | -1.012 | 0.010 |  |  |  |  |
|  | *HSPA8* | -0.912 | 0.032 |  |  |  |  |
|  | *ITPR1* | -0.941 | 0.040 |  |  |  |  |
|  | *KCNJ6* |  |  | 1.269 | 0.014 |  |  |
|  | *MAPK10* | -1.510 | 0.003 |  |  |  |  |
|  | *MAPK11* |  |  | 0.594 | 0.002 |  |  |
|  | *NPTN* | -1.129 | 0.038 |  |  |  |  |
|  | *NRG1* | 0.580 | 0.030 |  |  |  |  |
|  | *NSF* | -0.972 | 0.028 |  |  |  |  |
|  | *PLCB1* | -0.989 | 0.023 |  |  |  |  |
|  | *PPM1E* |  |  | 0.594 | <0.001 |  |  |
|  | *PPP1CA* | -0.573 | 0.035 |  |  |  |  |
|  | *PPP3R1* | -0.767 | 0.038 |  |  |  |  |
|  | *PRKAB2* |  |  |  |  | 3.165 | 0.039 |
|  | *PRKAG2* |  |  |  |  | 3.165 | 0.039 |
|  | *PRKCB* | -0.836 | 0.040 |  |  |  |  |
|  | *SLC17A7* | -1.418 | 0.001 |  |  |  |  |
|  | *SLC1A2* | -0.853 | 0.018 |  |  |  |  |
|  | *SNAP25* | -1.226 | <0.001 |  |  |  |  |
|  | *SYT1* | -1.206 | <0.001 |  |  |  |  |
|  | *TSPAN7* | -1.052 | 0.031 |  |  |  |  |
| **ADHD MAPK** | *ABL2* | -1.045 | 0.007 |  |  |  |  |
|  | *ACTN2* |  |  | -0.326 | 0.0167 |  |  |
|  | *AKT3* | 0.647 | 0.019 |  |  |  |  |
|  | *ATF2* | -1.198 | 0.045 |  |  |  |  |
|  | *BAD* | 0.738 | 0.006 |  |  |  |  |
|  | *DUSP5* |  |  | 1.055 | 0.003 |  |  |
|  | *DUSP6* |  |  | 0.736 | 0.003 |  |  |
|  | *FGF2* | 0.977 | <0.001 |  |  |  |  |
|  | *FRS2* |  |  |  |  | -2.519 | 0.039 |
|  | *GNAO1* | -0.895 | 0.029 |  |  |  |  |
|  | *GNG11* |  |  | -0.679 | 0.005 |  |  |
|  | *GNG3* |  |  | 0.317 | 0.035 |  |  |
|  | *GRB2* |  |  | 0.876 | 0.032 |  |  |
|  | *GRIN2A* |  |  | 0.306 | 0.036 |  |  |
|  | *HSPA8* | -0.912 | 0.032 |  |  |  |  |
|  | *IL17RD* | -1.489 | 0.006 |  |  |  |  |
|  | *IL2RG* |  |  |  |  | 2.466 | 0.039 |
|  | *MAPK1* |  |  | 0.207 | 0.039 |  |  |
|  | *MAPK10* | -1.510 | 0.003 |  |  |  |  |
|  | *MAPK11* |  |  | 0.594 | 0.002 |  |  |
|  | *NRG1* | 0.580 | 0.030 |  |  |  |  |
|  | *PLCG1* | 0.706 | 0.022 |  |  |  |  |
|  | *PPM1A* | -0.946 | 0.009 |  |  |  |  |
|  | *PPP3R1* | -0.767 | 0.038 |  |  |  |  |
|  | *PRKCB* | -0.836 | 0.040 |  |  |  |  |
|  | *RASGRP2* |  |  | -0.341 | 0.010 |  |  |
|  | *SPRED1* | -1.219 | 0.012 |  |  |  |  |
|  | *SPTBN1* | -0.761 | 0.023 |  |  |  |  |
|  | *TGFA* |  |  | -0.304 | 0.038 |  |  |
|  | *VCL* | -1.131 | 0.001 |  |  |  |  |

b)

| **Network** | **Gene ID** | **NAC** | | **NASH** | | **THY1** | |
| --- | --- | --- | --- | --- | --- | --- | --- |
|  |  | **Log_FC** | **Padj** | **Log_FC** | **Padj** | **Log_FC** | **Padj** |
| **ND Neuro-transmission** | *ACTN2* |  |  | 5.106 | 0.001 |  |  |
|  | *ADCY8* |  |  |  |  | -2.271 | 0.027 |
|  | *AKT2* | 4.035 | 0.028 |  |  |  |  |
|  | *ALDH2* |  |  | 2.098 | 0.024 |  |  |
|  | *ALDH5A1* |  |  |  |  | -0.987 | 0.05 |
|  | *AP2S1* |  |  | 2.954 | 0.025 |  |  |
|  | *CALM2* |  |  | 1.246 | 0.029 |  |  |
|  | *CALM4* | -4.322 | 0.025 |  |  |  |  |
|  | *DLG3* |  |  | 3.927 | 0.009 |  |  |
|  | *DLG4* |  |  | 1.61 | 0.032 |  |  |
|  | *GABRA5* |  |  |  |  | -0.597 | 0.02 |
|  | *GAD1* |  |  | 1.993 | 0.013 |  |  |
|  | *GLRB* |  |  | -0.718 | 0.034 |  |  |
|  | *GLUL* |  |  | 1.609 | 0.019 |  |  |
|  | *GNAI2* |  |  | 1.744 | 0.025 | 0.789 | 0.048 |
|  | *GNB1* |  |  |  |  | -1.059 | 0.005 |
|  | *GNG5* |  |  |  |  | 0.814 | 0.037 |
|  | *GRKI5* |  |  | 1.136 | 0.029 |  |  |
|  | *GRIN2A* | 3.556 | 0.028 |  |  |  |  |
|  | *HSPA8* |  |  | 2.323 | 0.005 |  |  |
|  | *MAPT* |  |  | 1.726 | 0.001 |  |  |
|  | *NCALD* |  |  |  |  | 0.819 | 0.031 |
|  | *PDPK1* |  |  |  |  | -0.547 | 0.047 |
|  | *PPFIA2* |  |  |  |  | -0.559 | 0.042 |
|  | *PPM1E* |  |  | 3.246 | 0.016 |  |  |
|  | *PPP1R1B* | 1.221 | 0.011 | 1.194 | 0.009 |  |  |
|  | *PPP2R2D* |  |  | 4.007 | 0.030 |  |  |
|  | *PRKACA* |  |  |  |  | -0.572 | 0.05 |
|  | *PRKAR1B* |  |  | 2.682 | 0.009 |  |  |
|  | *SLC6A11* |  |  |  |  | -3.83 | 0.035 |
|  | *VAMP2* |  |  | 1.7 | 0.005 |  |  |
| **ND MAPK** | *ABHD17A* |  |  | 1.956 | 0.046 |  |  |
|  | *ACTB* |  |  | 1.841 | 0.010 |  |  |
|  | *ACTN2* |  |  | 5.106 | 0.001 |  |  |
|  | *AKT2* |  |  | 4.035 | 0.028 |  |  |
|  | *ARAF* |  |  | -0.273 | 0.031 |  |  |
|  | *CACNA1H* |  |  | 2.606 | 0.013 |  |  |
|  | *CALM2* |  |  | 1.246 | 0.029 |  |  |
|  | *CSF1* |  |  | 4.193 | 0.023 |  |  |
|  | *DLG3* |  |  | 3.927 | 0.009 |  |  |
|  | *DLG4* |  |  | 1.61 | 0.032 |  |  |
|  | *DUSP4* | 1.361 | 0.007 |  |  |  |  |
|  | *DUSP8* |  |  |  |  | -0.633 | 0.042 |
|  | *FLNA* |  |  | 3.999 | 0.017 |  |  |
|  | *GNAI2* |  |  | 1.744 | 0.025 |  |  |
|  | *GNB1* |  |  |  |  | -0.806 | 0.002 |
|  | *GOLGA7* |  |  | 3.896 | 0.012 |  |  |
|  | *HSPA8* |  |  | 2.323 | 0.005 |  |  |
|  | *KBTBD7* |  |  |  |  | -1.255 | 0.032 |
|  | *MAP4K4* |  |  | 1.671 | 0.029 |  |  |
|  | *MAPK8IP1* |  |  | 1.407 | 0.031 |  |  |
|  | *MAPT* |  |  | 1.726 | 0.001 |  |  |
|  | *MET* |  |  |  |  | -1.224 | 0.000 |
|  | *NF1* |  |  |  |  | -0.792 | 0.025 |
|  | *NTRK2* | 4.905 | 0.003 |  |  |  |  |
|  | *PDE6D* |  |  |  |  | 1.071 | 0.013 |
|  | *PRKCZ* |  |  | 1.557 | 0.009 |  |  |
|  | *PSMB6* |  |  |  |  | 0.457 | 0.033 |
|  | *PSMC4* |  |  | 2.645378 | 0.046952 |  |  |
|  | *PSMC5* |  |  | 3.710429 | 0.00086 |  |  |
|  | *PSMD1* |  |  | 2.360597 | 0.010615 |  |  |
|  | *PSMD12* |  |  | 2.600562 | 0.073694 |  |  |
|  | *PSMD13* |  |  | 3.685298 | 0.024948 |  |  |
|  | *PSMD14* |  |  | 4.695564 | 0.003626 |  |  |
|  | *PTPRA* |  |  | 2.903 | 0.011 |  |  |
|  | *RAP1GAP* |  |  | 1.222 | 0.032 |  |  |
|  | *SPTAN1* |  |  | 1.189 | 0.003 |  |  |
|  | *SPTBN4* |  |  | 1.024 | 0.046 |  |  |
|  | *TYK2* |  |  |  |  | 4.289 | 0.023 |
|  | *UBA52* |  |  | 1.293 | 0.005 |  |  |
|  | *UBB* |  |  | 1.386 | 0.003 |  |  |
|  | *VFW* |  |  | 3.656 | 0.016 |  |  |
|  | *WDR83* | -0.318 | 0.042 |  |  |  |  |
|  | *YWHAB* |  |  | 1.812 | 0.02 |  |  |

*Supplemental Table 4*: Table of genes contributing to the enrichment of pathways mapped in Cytoscape for a) ADHD models and b) ND models. Genes are represented in rows and the corresponding log fold change (LogFC) and adjusted p-value (Padj) statistics are represented in columns according to which model they appeared in.

Differential gene expression and pathway enrichment analysis data is available upon request.
